# The effects of individual, family and environmental factors on physical activity levels in children: a cross-sectional study

**DOI:** 10.1186/1471-2431-14-107

**Published:** 2014-04-21

**Authors:** Sharon L Cadogan, Eimear Keane, Patricia M Kearney

**Affiliations:** 1 Department of Epidemiology and Public Health, University College Cork, Fourth Floor, Western Gateway Building, Western Road, Cork, Ireland

**Keywords:** Physical activity levels, Active, Children, Determinants, Predictors, Individual, Family, Environmental, School

## Abstract

**Background:**

Physical activity plays an important role in optimising physical and mental health during childhood, adolescence, and throughout adult life. This study aims to identify individual, family and environmental factors that determine physical activity levels in a population sample of children in Ireland.

**Methods:**

Cross-sectional analysis of the first wave (2008) of the nationally representative Growing Up in Ireland study. A two-stage clustered sampling method was used where national schools served as the primary sampling unit (response rate: 82%) and age eligible children from participating schools were the secondary units (response rate: 57%). Parent reported child physical activity levels and potential covariates (parent and child reported) include favourite hobby, total screen time, sports participation and child body mass index (measured by trained researcher). Univariate and multivariate multinomial logistic regression (forward block entry) examined the association between individual, family and environmental level factors and physical activity levels.

**Results:**

The children (N = 8,568) were classified as achieving low (25%), moderate (20%) or high (55%) physical activity levels. In the fully adjusted model, male gender (OR 1.64 [95% CI: 1.34-2.01]), having an active favourite hobby (OR 1.65 [95% CI: 1.31-2.08]) and membership of sports or fitness team (OR 1.90 [95% CI: 1.48-2.45]) were significantly associated with being in the high physical activity group. Exceeding two hours total screen time (OR 0.66 [95% CI: 0.52-0.85]), being overweight (OR 0.41 [95%CI: 0.27-0.61]; or obese (OR 0.68 [95%CI: 0.54-0.86]) were significantly associated with decreased odds of being in the high physical activity group.

**Conclusions:**

Individual level factors appear to predict PA levels when considered in the multiple domains. Future research should aim to use more robust objective measures to explore the usefulness of the interconnect that exists across these domains. In particular how the family and environmental settings could be useful facilitators for consistent individual level factors such as sports participation.

## Background

Physical activity (PA) plays a fundamental role in maintaining and improving physical and mental health, both during childhood and in later years [1,2]. Participating in high levels of PA during childhood produces immediate and, long-term health benefits in adulthood [3,4]. Despite the known health benefits, PA levels decline across the lifespan, particularly during adolescence [3,5-7]. Identified as the fourth leading risk factor for global mortality [8], physical inactivity is a major public health concern worldwide, associated with an estimated one million deaths annually in the World Health Organisation (WHO) European region alone [9].

WHO international guidelines recommend that children participate in at least 60 minutes of moderate-to-vigorous physical activity (MVPA) daily [10,11]. Worldwide, research has indicated that children are not achieving these guidelines, with estimates of activity levels varying both between and within countries [12-16]. For example, 42% of children aged six to 11 years in the United States [16] participate in 60 minutes of MVPA daily. Similarly, in the United Kingdom (UK), objectively measured PA measurements indicate that just 51% of four to 10 year olds (33% of four to 15 year olds) meet the recommended guidelines [12]. In comparison, 19% of primary school children and 14% of 10 to 18 year olds in Ireland meet the recommendations [14].

Achieving the recommended levels of PA per day is essential for the prevention and treatment of many health problems such as obesity. In particular, with evidence of tracking PA from childhood through adolescence and into adulthood [3], developing an active lifestyle from a young age may also produce long term benefits. However, to design effective strategies for increasing children’s PA levels, effects on, and determinants of, activity levels need to be well understood.

In order to structure relevant determinants, the conceptual framework for this research adopted Bronfenbrenner's ecological model of child development and well-being [17,18]. This model proposes that a child's development is affected by multiple levels of influencers including the micro-system which includes direct influencers such as family, school and neighbourhood [18]. Bronfenbrenner's model advocates the need to address factors at multiple levels in order to understand and change PA behaviours. Multilevel approaches derived from such ecological models have been recommended to examine PA determinants [19].

Existing evidence on correlates of PA in children have been reviewed extensively in the literature [7,20]. However, despite the awareness of multi-level associations, many of these factors have been investigated individually. Further, in 2009, the top five future research priorities for understanding and eliminating disparities in obesity, diet, and PA were published following a meeting of experts in the US [21]. One key recommendation for PA research was to “define individual and environmental factors using mixed methods and other new models to study both simultaneously” [21]. This research uses nationally representative data to examine the multi-level predictive capability of these correlates, specifically; the individual, family, and environmental level factors of PA among nine year olds in Ireland. The first aim of this study is to identify the distribution of individual, family and environmental factors by PA levels. A further novel objective is to model the multi-level effects of these factors on the PA levels of children at age nine.

## Methods

### Study design and sample

The sample comprised of 8,568 nine-year-old school-children participating in the first wave (2008) of Growing Up in Ireland (GUI) study [22]. GUI is a nationally representative cohort of nine year old children living in the Republicof Ireland. The data (in the form of an Anonymised Microdata File, AMF) are archived in the Irish Social Science Data Archive (ISSDA) and are available to researchers on request.

Eligibility criteria included children who were born between 1st November 1997 and 31st October 1998. The sample was selected using a two-stage clustered sampling method within the Irish primary school system (all mainstream, special and private schools), whereby the school was the primary sampling unit and the age eligible children attending the school were the secondary units [23,24]. In the first stage, 1,105 schools from the national total of 3,200 were randomly selected using probability proportionate to size sampling, followed by recruitment of a random sample of eligible children within each school (stage two). At the school level, a response rate of 82.3% (910 schools) was achieved, while at the level of the household (i.e. eligible child) 57% of children and their parent/guardians participated in the study. Fieldwork for the school-based component was carried out between March-November 2007, while fieldwork for the home-based phase of data collection ran from July 2007-July 2008. The data were weighted prior to analysis to account for the complex sampling design, which involves the structural adjustment of the sample to the population using Census of Population statistics while maintaining the case base of 8,568 children. More detailed information on the sampling, data collection process and derivation of weights is available elsewhere [24].

Ethical approval was granted by the Health Research Board’s Research Ethics Committee based in Dublin, Ireland. Written informed consent was also obtained from a parent or guardian and the study child prior to commencement of the data collection process [24].

### Data collection procedures

Trained social interviewers conducted interviews with the study child and their primary caregiver (and second parent/guardian where applicable) within the home. Parents nominated a primary caregiver (the parent who spent most time with the study child) who was the primary respondent. In 98% of cases, this was the study child’s biological mother. The main interviews were completed on a Computer Assisted Personal Interview (CAPI) basis. There was also a self-complete paper-based supplement for all respondents, which included some potentially sensitive questions such as issues about the marital relationship, marital conflict, experience of depression, and use of drugs [24]. Sources and validity of each of the questions used for this research is contained elsewhere [24]. Anthropometric measurements for the primary and secondary caregiver as well as the study child were also taken during the household interview using standard procedures [24].

### Dependent variable

Child PA levels were calculated separately using data reported by the study child’s primary caregiver (mother for 98% of children). The PA questions included in the primary caregivers questionnaire were adapted from the Leisure Time Exercise Questionnaire [25]. The caregiver reported *the number of days out of the previous 14 that the child had engaged in ‘hard’ exercise for at least 20 minutes*. Hard exercise was defined as exercise that resulted in heavy breathing and a fast heart beat [23]. This self-report measure has been shown to demonstrate concurrent validity with measures of maximum oxygen intake (VO2 max) and muscular endurance [26], as well as acceptable test-retest reliability [27].

Study child’s PA was re-coded into a three level variable based on previous research [28]: low “0-4 days”, moderate “5-8 days” and high “>9 days” PA groups. Nine or more days out of previous 14 was the highest possible value and corresponds closest to the recommended PA guidelines. This is also consistent with other Irish research using the same wave of the GUI data [29].

### Covariates

#### Individual level variables

Five individual level variables were included: the study child’s gender, whether the study child was a member of a sports or fitness club (yes/no), total screen time (TST [<2 hours TST per day/>2 hours TST per day]), the nature of study child’s favourite hobby (active/inactive) and the study child’s weight status (normal/overweight/obese). Data for the former three variables was primary caregiver reported. The study child’s favourite hobby variable was based on child reported data. Weight status was classified using objectivity measured data.

TST was categorised based on the recommendations of the American Academy of Paediatricians [30]. This variable was created by combining three screen time variables; hours spent watching TV/videos, playing video games and using a computer (<1 hour, 1-3hours, >3 hours). This resulted in a seven level response variable, classified as: “adhering to (<) the recommended maximum two hours/day” or “exceeding the recommended two hours/day”. Adhering to the recommended TST was defined as, the study child only exceeding one hour of screen time in one of the screen time variables (giving a potential for maximum two hours TST).

The study child’s favourite hobby variable was created using 32 hobbies listed by the child, classified into a two level response “active” or “inactive” (16 hobbies in each group). A hobby was considered active if it required the child having a physically active participatory role and inactive if the child had a permissive role or remained sedentary. Active hobbies included: basketball, football, hockey and gymnastics. Inactive hobbies included: reading, listening to music and watching TV.

Trained interviewers were responsible for height and weight measurements of each study child and each adult respondent. Height data was recorded to the nearest millimetre using a Leicester portable height stick [24]. Weight was recorded using a SECA 761 flat mechanic scales to the nearest 0.5 kilogram [24]. Children’s body mass index (BMI) were classified as normal weight, overweight (BMI of 19.46 for boys and 19.45 for girls) or obese (BMI of 23.39 for boys and 23.46 for girls) using age (9.5 years) and gender specific International Obesity Taskforce (IOTF) cut off points [31].

#### Family level variables

Six family level variables were included: primary caregiver’s education (third level/post-secondary/ higher secondary/lower secondary or less), employment status (in full time work/ not in full time work), parenting style (authoritative/permissive) primary caregiver weight status (normal, overweight or obese), whether the child has siblings (yes/no) and the household structure (two parent/single parent). These variables were based on primary caregiver reported data with the exception of objectively measured weight status.

The parenting style variable described the practices of the child’s primary caregiver. For the purpose of this research, the original GUI responses; authoritarian, authoritative, permissive and uninvolved parenting styles were re-coded as “authoritative” or “permissive”. The primary caregiver’s measured BMI data was classified according to WHO guidelines as normal weight (<25 kg/m^2^), overweight (≥25 and <30 kg/m^2^) or obese (≥30 kg/m^2^) [32].

#### Environmental level variables

Five environmental level variables were included: transport to and from school (active/both active and inactive/inactive), school playground (good or excellent/fair or poor), school sports facilities (good or excellent/fair or poor), after school activities (agree/disagree) and safe play areas in neighbourhood (agree/disagree).

The school transport variable (caregiver reported) was created using questions on how the study child travelled both to and from school (walks, by public transport, school bus/coach, car, cycles or other). Responses were combined and re-coded as “active both ways”, “active one way, inactive one way” and “inactive both ways”.

The school playground and sports facilities data were obtained from the school principal questionnaire while data on neighbourhood facilities were primary caregiver reported. Responses for school facilities were re-coded as “very good/excellent” or “fair/poor”. Responses to both neighbourhood facilities were re-coded as “agree” or “disagree”.

### Statistical analysis

Secondary analysis was performed using stata (version 12, intercooled). P-values less than 0.05 were considered statistically significant. Probability weights were applied to the data using survey data commands to account for the complex survey design.

Missing data levels were very low for the majority of the variables used, and where missing values were identified (e.g. 5.2% of PCG BMI measurements) it was found not to be missing at random and hence, data could not be imputed. Primary caregiver reported PA data was available for 99.9% of the study children, giving an effective case base of 8,566 children for analysis.

Descriptive statistics were performed to evaluate the children’s PA related characteristics. Unadjusted multinomial logistic regression methods were used to measure the association between independent predictor variables and moderate/high PA levels. Multinomial multivariate logistic regression was conducted to assess their predictive capability (adjusting for all potential confounders) using the forward block entry function: individual, family and environmental blocks. The first block (model one) included the five individual level factors: gender, weight status, TST, favourite hobby and being a member of a sports or fitness team. Block two (model 2) included the six family level factors: primary caregiver’s education, primary caregiver’s employment status, primary caregiver’s weight status, siblings, parenting style and household structure. Block three (model 3) contained the five environmental level factors: transport to and from school, school’s playground facilities, school sports facilities, safe neighbourhood to play in and after school activities.

## Results

### Overview of children’s PA patterns

Children were categorised into three PA groups: low (N = 2,135), moderate (N = 1,740) and high (N = 4,691). Overall, 26.3% (95% CI, 24.9-27.7) had low, 19.3% (95% CI, 18.2-20.5) had moderate and 54.4% (95% CI, 52.8-55.9) had high PA levels. Gender differences existed, with 61% (N = 2,609) of boys categorised as being highly active (high PA group) compared to 48% (N = 2,082) of girls (p < 0.001). PA/obesity related demographics stratified by gender are presented in Table 1. Over half of the children (N = 4,730) reported taking exercise almost every day (55% of boys vs. 45% of girls, p < 0.001), of which 65% (N = 3,123) were in the high, 16% (N = 794) in the moderate and 19% (N = 813) in the low PA groups (p < 0.001). According to child reported data, 25% (N = 2,136) of children met the WHO guidelines of participating in 60 minutes of MVPA each day. Boys were more likely to achieve the recommended guideline than girls (29% versus 21%, p < 0.001). Valid height and weight measurements for the study child were also obtained for 94.5% (N = 8,136) of the sample. The estimated proportion of children in the normal, overweight, and obese categories was 74.1% (95% CI, 72.8-75.3), 19.3% (95% CI, 18.2-20.5) and 6.6% (95% CI, 5.9-7.4), respectively.

**Table 1:** Physical activity/obesity related characteristics of the children by gender and PA levels

		**Boys**					**Girls**			
		**Low PA**	**Moderate PA**	**High PA**			**Low PA**	**Moderate PA**	**High PA**	
		**(N = 826)**	**(N = 728)**	**(N = 2,609)**			**(N = 1,309)**	**(N = 1,012)**	**(N = 2,082)**	
**Individual factors**^+^	**Total**	**N (%)**	**N (%)**	**N (%)**	**p-value**	**Total**	**N (%)**	**N (%)**	**N (%)**	**p-value**
Child’s weight status**					<0.001					<0.001
Normal	3,100	558 (20)	541 (17)	2,001 (63)		3,019	812 (27)	692 (22)	1515(51)	
Overweight	661	137 (21)	115 (17)	409 (62)		875	296 (36)	204 (21)	375 (43)	
Obese	196	74 (39)	39 (18)	83 (43)		284	122 (48)	65 (20)	97 (33)	
Takes exercise					<0.001					<0.001
Never	34	22 (62)	4 (10)	8 (28)		44	25 (69)	7 (10)	12 (21)	
1-2times/week	673	243 (38)	144 (20)	286 (42)		957	446 (47)	223 (21)	288 (32)	
3-4times/week	939	209 (25)	234 (25)	496 (51)		1,136	356 (33)	329 (27)	453 (40)	
Almost every day	2,486	341 (16)	344 (14)	1801 (70)		2,244	472 (22)	450 (19)	1,322(59)	
Sports/fitness club					<0.001					<0.001
Yes	3,585	596 (18)	644 (18)	2345 (64)		3,137	809 (26)	768 (24)	1,560 (49)	
No	573	226 (41)	84 (14)	263 (45)		1,261	496 (40)	244 (16)	521 (44)	
Playing sport*					<0.001					0.11
Favourite ^#^	1,657	232 (15)	258 (16)	1167 (69)		809	178 (23)	195 (24)	436 (53)	
Second favourite	968	155 (17)	187 (19)	636 (64)		767	207 (30)	179 (22)	381 (48)	
Third favourite	455	97 (27)	78 (16)	280 (57)		506	144 (31)	123 (22)	239 (48)	
Watching TV*					0.38					0.99
Favourite	169	53(29)	33 (19)	83 (52)		195	73 (35)	42 (20)	80 (45)	
Second favourite	491	126 (28)	88 (20)	277 (53)		428	144 (34)	94 (21)	190 (45)	
Third favourite	669	135 (23)	128 (18)	406 (59)		551	187 (35)	121 (21)	243 (44)	
Playing video games*					<0.001					0.36
Favourite	211	71 (37)	42 (17)	98 (46)		84	34 (35)	22 (29)	28 (36)	
Second favourite	318	84 (30)	64 (19)	170 (51)		202	61 (28)	54 (24)	87 (48)	
Third favourite	392	83 (21)	70 (18)	239 (62)		255	86 (36)	58 (20)	111(43)	
Watching TV					<0.001					<0.001
Zero or <1 hour	1,050	157 (17)	174 (15)	719 (68)		1,186	274 (25)	279 (21)	633 (54)	
1-3hours	2,723	539 (21)	475 (18)	1,709 (62)		2,819	856 (30)	656 (22)	1,307 (47)	
>3 hours	390	130 (35)	79 (21)	181 (44)		398	179 (45)	77 (19)	142 (36)	
Playing video games					<0.001					0.02
Zero or <1 hour	3,059	549 (20)	522 (17)	1,988 (63)		3,923	1,118 (29)	923 (22)	1,882 (48)	
1-3hours	1,011	245 (26)	185 (17)	581 (57)		438	169 (37)	82 (16)	187 (47)	
>3 hours	93	32 (38)	21 (21)	40 (41)		39	19 (46)	7 (21)	13 (32)	
On the computer					<0.001					0.34
Zero or <1 hour	3,650	669 (21)	624 (17)	2,337 (62)		3,820	1,097 (30)	895 (22)	1,828 (48)	
1-3hours	498	143 (31)	94 (17)	261 (52)		549	200 (35)	112 (44)	237 (44)	
>3 hours	33	14 (33)	9 (31)	10 (36)		32	11 (31)	5 (15)	16 (53)	
Total screen time					<0.001					0.002
<2 hours/day	899	128 (17)	142 (14)	629 (69)		1,082	247 (25)	258 (22)	577 (53)	
>2 hours/day	3,262	698 (23)	585 (18)	1,979 (59)		3,317	1,059 (32)	754 (22)	1,504 (46)	
**Family factors**^+^										
Caregiver’s weight***					0.74					0.13
Normal	1,925	349 (21)	340 (17)	1,236 (62)		1,962	552 (29)	466 (21)	944 (50)	
Overweight	1,244	262 (22)	224 (18)	758 (60)		1,300	391 (31)	282 (21)	627 (47)	
Obese	655	149 (23)	112 (17)	394 (60)		1735	248 (35)	172 (23)	315 (43)	
Caregiver’s education					0.01					<0.001
</=lower second level	674	156 (25)	108 (15)	410 (60)		834	281 (35)	151 (17)	402 (48)	
Higher second level	1,295	287 (23)	248 (19)	760 (58)		1,403	428 (31)	319 (22)	656 (47)	
Post second level	1,056	203 (21)	173 (16)	680 (63)		1,067	302 (27)	241 (23)	524 (50)	
Third level	1,138	180 (17)	199 (17)	759 (66)		1,099	298 (26)	301 (27)	500 (47)	
Siblings					0.59					0.17
Yes	3,716	728 (22)	656 (18)	2,332 (61)		3,977	1,166 (30)	910 (21)	1,901 (49)	
No	329	66 (20)	57 (16)	206 (64)		330	101 (32)	88 (26)	141 (42)	
Household type					0.01					0.71
Single Parent	457	119 (28)	58 (14)	280 (59)		534	165 (32)	118 (20)	251 (48)	
Two parent	3,706	707 (21)	670 (18)	2,329 (61)		3,869	1,144 (31)	894 (22)	1,831 (47)	
**Environmental factors**^+^										
Transport to school					0.21					0.68
Active	1,047	199 (23)	167 (15)	681 (62)		1,085	298 (30)	261 (22)	526 (49)	
Inactive	3,116	627 (22)	561 (18)	1,928 (60)		3,318	1,011 (31)	751 (21)	1,556 (47)	
Transport from school					0.24					0.48
Active	1,160	223 (23)	190 (15)	747 (63)		1,209	339 (30)	298 (23)	572 (48)	
Inactive	3,003	603 (21)	538 (18)	1,862 (60)		3,189	967 (31)	713 (21)	1,509 (48)	
School playground^					0.19					0.79
Fair/poor	1,660	320 (22)	316 (19)	1,024 (59)		1,704	501 (30)	398 (22)	805 (48)	
Good/excellent	2,361	453 (22)	389 (16)	1,489 (62)		2,493	751 (31)	573 (21)	1,169 (48)	
School sports facilities^					0.97					0.43
Fair/poor	1,765	343 (22)	310 (17)	1,112 (60)		1,908	573 (31)	417 (20)	918 (48)	
Good/excellent	2,267	460 (22)	397 (17)	1,410 (61)		2,341	698 (31)	563 (22)	1080 (47)	
Safe places to play					0.34					0.18
Agree	3,814	740 (22)	662 (17)	2412 (61)		4,016	1,194 (30)	925 (22)	1,897 (48)	
Disagree	344	85 (26)	64 (16)	195 (58)		113	381 (36)	87 (19)	181 (46)	

^+^All data is primary caregiver reported unless indicated otherwise.

*Child-reported variable.

**Weight status defined as BMI classified according to International Obesity Taskforce on Obesity age and gender specific guidelines using objectively measured height and weight data.

***Weight status defined as BMI classified according to World Health Organisation guidelines using objectively measured height and weight data.

^School principal reported data.

^#^Favourite refers to the study child reporting the hobby as being their favourite thing to do.

### Univariate logistic regression findings

Table 2 presents the results of the univariate multinomial logistic regression. All five of the individual level factors were found to be associated with high PA while four were found to be associated with moderate PA levels. Of the family level factors, primary caregiver’s education, primary caregiver’s employment status, household structure and parenting style were significantly associated with moderate PA levels, while having siblings and primary caregiver’s weight status were not. None of the school level factors were associated with either moderate or high PA levels, while, both safe playgrounds and participating in after school activities in the children’s neighbourhood were found to be associated with both moderate and high PA.

**Table 2:** Independent association of each of the individual, family and environmental level factors and moderate or high PA levels

**Variable**	Moderate PA* (N = 1,740)		High PA* (N = 4,691)	
	**OR (95% CI)**	**p-value**	**OR (95% CI)**	**p-value**
**Individual factors**^+^				
Gender				
Boys	1.13 (0.96-1.33)	0.14	1.79 (1.55-2.07)	<0.001
Girls	1***		1	
Child’s weight status^#^				
Obese	0.52 (0.38-0.71)	<0.001	0.34 (0.26-0.44)	<0.001
Overweight	0.78 (0.64-0.95)	0.01	0.71 (0.60-0.84)	<0.001
Normal	1		1	
Child’s favourite hobby**				
Active hobby^	1.26 (1.08-1.48)	0.01	1.81 (1.57-2.08)	<0.001
Inactive hobby	1		1	
Sports/fitness club				
Yes	2.49 (2.09-2.98)	<0.001	2.41 (2.0-2.82)	<0.001
No	1		1	
Total screen time				
<Recommended 2 hours	0.83 (0.67-1.01)	0.06	0.66 (0.56-0.78)	<0.001
>Recommended 2 hours	1		1	
**Family factors**^+^				
Caregiver’s employment				
Not in full time	1.31 (1.02-1.69)	0.04	1.23 (0.99 -1.53)	0.06
In full time work	1		1	
Caregiver’s education				
Third level	1.93 (1.51-2.46)	<0.001	1.56 (1.28-1.90)	<0.001
Post-secondary	1.48 (1.17-1.87)	<0.001	1.35 (1.13-1.62)	0.001
Higher secondary	1.45 (1.16-1.79)	<0.001	1.10 (0.94-1.32)	0.23
< =Lower secondary	1		1	
Caregiver’s weight status^##^				
Obese	0.86 (0.68-1.09)	0.21	0.77 (0.64-0.94)	0.01
Overweight	0.96 (0.79-1.17)	0.69	0.89 (0.76-1.04)	0.13
Normal	1		1	
Siblings				
Yes	0.92 (0.67-1.25)	0.57	1.02 (0.78-1.32)	0.90
No	1		1	
Household type				
Two parent	1.37 (1.07-1.75)	0.01	1.19 (0.99-1.45)	0.06
Single parent	1		1	
Parenting style				
Authoritative	1.26 (1.03-1.55)	0.02	1.22 (1.03-1.44)	0.02
Permissive	1		1	
**Environmental factors**^+^				
Travel to/from school				
Active both ways	0.94 (0.77-1.15)	0.54	1.02 (0.87-1.20)	0.80
Active one way	1.12 (0.83-1.52)	0.46	1.00 (0.79-1.28)	0.99
Inactive both ways	1		1	
School playground***				
Good/excellent	0.89 (0.74-1.06)	0.18	1.00 (0.86-1.18)	0.94
Fair/poor	1		1	
School sports facilities***				
Good/excellent	1.08 (0.91-1.28)	0.37	1.03 (0.88-1.20)	0.75
Fair/poor	1		1	
Safe places to play				
Agree	1.20 (1.02-1.41)	0.03	1.20 (1.05-1.38)	0.01
Disagree	1		1	
After school activities				
Yes	1.29 (1.07-1.56)	0.01	1.22 (1.04-1.42)	0.01
No	1		1	

^+^All data is primary caregiver reported unless indicated otherwise.

*Reference category: low PA.

**Child-reported data.

***School principal reported data.

****1 denotes reference category.

^Active hobby was defined as one in which the study child had a physically active participatory role.

^#^Weight status defined as BMI classified according to International Obesity Taskforce on Obesity age and gender specific guidelines using objectively measured height and weight data.

^##^Weight status defined as BMI classified according to World Health Organisation guidelines using objectively measured height and weight data.

### Multivariate logistic regression findings

**Figure 1:**
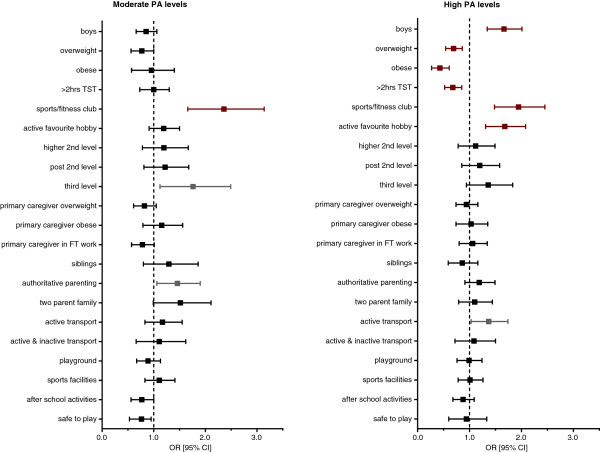
Individual, family and environmental factors associated with moderate and high physical activity.

Figure 1 illustrates the findings (final model) of the multivariate multinomial logistic regression analyses.

### Model one (individual level factors)

Of the individual level factors, male gender (p < 0.001), having a physically active favourite hobby (p < 0.001) and being a member of a sports or fitness group (p < 0.001) were positively associated with high PA levels (Table 3). Being a member of a sports or fitness team (p < 0.001) was positively associated with moderate PA. Being overweight or obese was negatively associated with both moderate and high PA, while exceeding the recommended maximum TST (two hours) was negatively associated with high PA (p < 0.001). Obese children were 60% and 42% less likely to be in the high and moderate PA groups, respectively (OR, 0.40 [95% CI, 0.31-0.52] p < 0.001; OR: 0.58 [95% CI: 0.42-0.79] p < 0.001) compared to children of normal weight. Overweight children were 21% and 23% less likely to be in the moderate and high PA groups, respectively (OR, 0.79 [95% CI: 0.65-0.97] p = 0.02; OR: 0.77 [95% CI, 0.64-0.91] p = 0.003). Children who exceeded two hours TST were 23% less likely to be in high PA group (OR, 0.71 [95% CI, 0.59-0.84] p < 0.001).

**Table 3:** Multivariate multinomial analysis of the individual, family and environmental factors and PA levels

	**Model 1 (individual level factors)**				**Model 2 (model 1 + family level factors)**				**Model 3 (model 1 and 2 + environmental factors)**			
	**Moderate**		**High**		**Moderate**		**High**		**Moderate**		**High**	
	**OR (95%CI)**	**p-value**	**OR (95%CI)**	**p-value**	**OR (95%CI)**	**p-value**	**OR (95%CI)**	**p-value**	**OR (95%CI)**	**p-value**	**OR (95%CI)**	**p-value**
**Individual factors**^+^												
Gender												
Boys	0.96 (0.81-1.14)	0.63	1.54(1.32-1.80)	<0.001	0.85 (0.68 -1.08)	0.19	1.66 (1.37-2.01)	<0.001	0.84 (0.66-1.06)	0.15	1.64 (1.34-2.01)	<0.001
Girls	1***		1		1		1		1		1	
Child’s weight status^#^												
Obese	0.58 (0.42-0.79)	<0.001	0.40 (0.31-0.52)	<0.001	0.86 (0.55-1.34)	0.51	0.41 (0.27-0.61)	<0.001	0.90 (0.57-1.40)	0.63	0.41 (0.27-0.61)	<0.001
Overweight	0.79 (0.65-0.97)	0.02	0.77 (0.64-0.91)	0.001	0.72 (0.55-1.34)	0.02	0.68 (0.54-0.85)	<0.001	0.75 (.56-1.00)	0.05	0.68 (0.54-0.86)	<0.001
Normal	1		1		1		1		1		1	
Total screen time^												
< Recommended 2 hours	0.90 (0.73-1.11)	0.33	0.71 (0.59-0.84)	<0.001	0.97 (0.73-1.28)	0.82	0.67 (0.53-0.86)	<0.001	0.97 (0.73-1.30)	0.85	0.66 (0.52-0.85)	<0.001
>Recommended 2 hours	1		1		1		1		1		1	
Favourite hobby*												
Active	1.13 (0.95-1.35)	0.27	1.65 (1.42-1.92)	<0.001	1.21 (0.95-1.53)	0.13	1.62 (1.30-2.03)	<0.001	1.17 (0.91-1.50)	0.21	1.65 (1.31-2.08)	<0.001
Inactive	1				1		1		1		1	
Sports/fitness club												
Yes	2.28 (1.88-2.77)	<0.001	1.86 (1.58-2.20)	<0.001	2.32 (1.69-3.18)	<0.001	1.92 (1.50-2.46)	<0.001	2.28 (1.66-3.14)	0.001	1.90 (1.48-2.45)	<0.001
No	1		1		1				1		1	
**Family factors**^+^												
Caregiver’s education												
Third level					1.74 (1.18-2.57)	0.01	1.32 (0.96-1.81)	0.09	1.67 (1.12-2.50)	0.01	1.31 (0.94-1.83)	0.11
Post-secondary					1.21 (0.85-1.72)	0.29	1.17 (0.87-1.57)	0.31	1.14 (0.78-1.67)	0.46	1.16 (0.85-1.58)	0.34
Higher secondary					1.17 (0.81-1.68)	0.41	1.09 (0.80-1.47)	0.60	1.16 (0.81-1.68)	0.42	1.08 (0.79-1.49)	0.62
< =Lower secondary					1		1		1		1	
Caregiver’s employment												
In full time work					0.81 (0.61-1.08)	0.15	1.05 (0.82-1.34)	0.73	0.76 (0.57-1.01)	0.06	1.04 (0.72-1.50)	0.79
Not in full time					1		1		1		1	
Caregiver’s weight^#^												
Obese					1.14 (0.82-1.58)	0.43	1.02(0.76-1.36)	0.90	1.11 (0.79-1.56)	0.54	1.00 (0.74-1.35)	0.99
Overweight					0.85 (0.65-1.11)	0.23	0.94 (0.75-1.17)	0.56	0.80 (0.61-1.05)	0.11	0.92 (0.74-1.16)	0.48
Normal					1		1		1		1	
Siblings												
Yes					1.20 (0.80-1.79)	0.37	0.86 (0.62-1.18)	0.35	1.22 (0.80-1.86)	0.35	0.83 (0.59-1.16)	0.28
No					1		1		1		1	
Parenting style												
Authoritative					1.41 (1.07-1.87)	0.02	1.15 (0.90-1.47)	0.25	1.42 (1.06-1.90)	0.02	1.16 (0.91-1.49)	0.23
Permissive					1		1		1		1	
Household type												
Two parent					1.38 (0.96-1.98)	0.08	1.03(0.77-1.38)	0.82	1.45 (0.99-2.12)	0.06	1.07 (0.79-1.44)	0.74
One parent					1		1		1		1	
**Environmental factors**^+^												
Travel to/from school												
Active both ways									1.13 (0.83-1.41)	0.43	1.34 (1.03-1.74)	0.03
Active one way									1.04 (0.66-1.62)	0.88	1.04 (0.72-1.50)	0.85
Inactive both ways									1		1	
School playground**												
Good/excellent									0.87 (0.67-1.62)	0.29	0.97 (0.76-1.24)	0.82
Fair/poor									1		1	
School sports facilities**												
Good/excellent									1.08 (0.83-1.41)	0.56	0.99 (0.78-1.26)	0.95
Fair/poor									1		1	
Safe places to play												
Agree									0.81 (0.53-1.24)	0.33	1.12 -0.75-1.66)	0.58
Disagree									1		1	
After school activities												
Yes									1.39 (1.05-1.84)	0.02	1.16 (0.92-1.46)	0.22
No									1		1	

^+^All data is primary caregiver reported unless indicated otherwise.

*Child-reported variable.

**School principal reported variable.

^#^Objectively measured height and weight data.

***1 denotes reference category.

^Screen time was according to the American Association of Pediatrics guidelines.

### Model two (individual and family level factors)

None of the family level factors were found to be associated with high PA. Primary caregivers having third level education and an authoritative parenting style were both positively associated with moderate PA levels (Table 3). Children who had primary caregivers with a third level degree were 1.74 times more likely to be in the moderate PA group compared to children of parents with a lower secondary education or less (OR 1.74 [95% CI: 1.18-2.57] p < 0.01). Having a primary caregiver who adopts an authoritative parenting style was associated with a 42% increase in the child’s probability of being in the moderate PA group (OR 1.42 [95% CI: 1.06-1.87] p = 0.02) compared to having a primary caregiver with a permissive parenting style.

In model two, the strength of the association for three of the significant individual level factors (gender, weight status and being a member of a sports or fitness team) became stronger. In particular, the probability of being in the high PA group was 66% higher for boys (OR: 1.66 [95% CI: 1.37-2.01] p < 0.01).

### Model three (fully adjusted model)

Accounting for both individual and family level factors, active travel to and from school was positively associated with high PA levels. A positive association between living in a neighbourhood with after school activities and moderate PA was also identified. Children who used active mode of travel both to and from school were 34% more likely to be in the high PA group (OR 1.34 [95% CI: 1.03-1.74] p = 0.03) compared to children who used an inactive mode of travel both to and from school. Children living in a neighbourhood with after school activities were 39% more likely to be in the moderate PA group compared to those who lived in neighbourhoods without after school activities (OR 1.39 [95% CI: 1.05-1.84] p = 0.02).

The association between the individual level factors and high PA remained statistically significant. Of the family level factors, having a primary caregiver with third level education and authoritative parenting styles remained positively associated with moderate PA levels. None of the family level factors were associated with high PA.

## Discussion

To our knowledge, this is the first study to explore the multi-level effects of individual, family and environmental factors on PA levels of children in Ireland. A key finding of this research is that individual level factors appear to have the strongest association with PA levels in nine year olds. Further, many of these factors are modifiable. Being a member of a sports or fitness club, and, having an active favourite hobby were both positively associated with higher levels of PA. Exceeding two hours of TST and being overweight or obese were negatively correlated with higher PA levels. No significant associations with the family level and just one marginal association among the environmental level factors were identified. However, environmental level factors could provide cost effective settings for implementing PA initiatives such as supporting sports participation.

Consistent with both extensive reviews by Sallis et al. [7] and van der Horst et al. [20], boys were more likely to have high PA levels. Literature suggests that differences in organised sports participation may be responsible for some of gender disparities in PA levels. In this research, over 75% of the children were members of a sports or fitness group (84% of boys versus 67% of girls, p = 0.000). In the fully adjusted model (controlled for gender), this research found children who were members of a sports or fitness group were almost twice as likely to be in the high PA group compared to children who were not. This is consistent with findings of the review by Sallis et al. which concluded that community sports participation [7] was positively associated with higher PA levels. Despite generally higher sports participation among boys, a review of PA correlates among girls aged between 10 and 18 also found that organised sports participation had a consistent positive association with higher PA levels [33]. Moreover, longitudinal studies have reported that participation in organised sports during childhood may be associated with long-term participation in PA in both adolescence and adulthood [3,34]. The promotion of sports and other high intensity activities may therefore provide an opportunity to increase PA among school children.

Many sports and other high intensity activities take place as extra-curricular activities after school hours. The Irish primary school day typically lasts five hours and 40 minutes, commencing at 9 am and finishing at approximately 3 pm. While the curriculum recommends one hour of physical education per week, it has been suggested that many schools do not provide this [14]. As a result, children’s preferences for extracurricular activities may also play a role in their overall PA levels. This research found that children reporting a preference for an active favourite hobby (including basketball, gymnastics and hockey) were more likely to be in the high PA group compared to children who preferred inactive favourite hobbies such as reading, listening to music, and watching TV. Similarly, in their review of previous research, Sallis et al. [7] concluded that children's preference for physical (rather than sedentary) activity was one of the factors most consistently associated with their participation in such activity.

Another key factor that may be associated with PA levels among nine year olds is sedentary behaviour. The American Academy of Paediatricians recommends that children do not exceed two hours of sedentary screen time per day [30]. Previous Irish research reported that over 99% of children and youth exceeded the recommended maximum two hours sedentary screen time per day [14]. Conflicting evidence exists for an association between sedentary behaviours (including screen time) and PA levels among children [7,35]. This present research found that exceeding these guidelines reduced the likelihood of high PA by 44%. The literature refers to the displacement theory as a possible explanation for an association between exceeding the recommended and lower PA, that is, sedentary behaviours may be replacing active behaviours [36].

PA behaviour and the factors influencing it are very complex. The social-ecological model adopted by this present research is a useful framework due to the complexity of behaviours [18]. Each level of the model layers (individual, family and environmental) is interconnected. Exploring the multiple domains, this present research has considered the broader context when identifying the predictors of PA. While this research did not identify environmental factors as major determinants of PA, more research is needed. In particular, the importance of built environments for increasing PA and other health behaviours has emerged in the literature [37,38]. Hence, applying the social-ecological theory, objective measures of PA, along with more robust environmental level factors should be considered for modelling PA.

This research used robust objectively measured data for calculating the child’s weight status. While two large reviews [7,20] have reported inconclusive or no relationship between weight status and PA levels, this research found that the weight status of the child was negatively associated with PA levels. Using objectively measured BMI data, being overweight or obese was associated with lower levels of PA. A possible explanation for this contrasting finding may be the use parent-reported height and weight data for children in other research, which has been found to lack validity and reliability when compared with objective anthropometric measures [39].

A key strength of this study is the large sample of nine year olds taken from the most comprehensive nationally representative children’s health survey currently available in Ireland. According to the 2006 Census figures, there were 56,497 nine year old children resident in Ireland [23]. Thus, this data includes approximately one seventh of these children. Further, probability weights were applied to the data using survey data commands to ensure that the findings are national representative.

However, there are some limitations to this study. The data analysed for this research is cross-sectional, therefore, a causal relationship cannot be inferred. The sample only included nine year old children, hence, generalisability cannot be assumed for all children. Also, there was a relatively low response rate at the household level (57%). The data has been weighted to overcome any issues arising from this; however, response bias may exist.

Further, the nature of the PA data collected does not correspond with WHO guidelines – 60 minutes of MVPA per day [10]. While self-report (by child) of the WHO PA levels was available, the data were not used for the dependent variable as it has been found that children under 10 are not reliable at recalling PA patterns, in particular PA intensity [40]. Validity studies have concluded that studies should use objective measures of PA, or if this is not feasible, rely on parental reports of child PA [41]. The PA data available for this research was primary caregiver reported as opposed to objectively measured data. The primary caregiver reported PA based on how many days in the last 14 the study child had achieved at least 20 minutes of hard physical activity. This self-report question was found to be reliable with acceptable validity when compared with accelerometer data [42]. Also, using this question, other Irish research has constructed PA categories in the same way [29]. Finally, this research provides a comprehensive list of individual level factors; however, some family and environmental level factors were not available such as the primary caregiver’s PA patterns.

## Conclusions

In conclusion, this study finds individual level factors; including many modifiable factors appear to have the strongest correlation with PA levels of nine year olds in Ireland. Remarkably, individual characteristics appear to predict PA levels when considered in the multiple domains. Future research should aim to use more robust objective measures to explore the usefulness of the interconnect that exists across these domains. In particular how the family and environmental settings could be useful facilitators for consistent individual level factors such as sports participation.

## Abbreviations

BMI: Body mass index; PA: Physical activity; MVPA: Moderate-to-vigorous physical activity; TST: Total screen time; WHO: World Health Organisation.

## Competing interests

The authors have indicated they have no conflicts of interest or financial relationships relevant to this article to disclose.

## Authors’ contributions

SC, EK and PK conceived the overall study design and SC analysed the data and drafted the initial manuscript. All authors have participated in the revising of the manuscript; and all have approved the manuscript as submitted.

## Pre-publication history

The pre-publication history for this paper can be accessed here:

http://www.biomedcentral.com/1471-2431/14/107/prepub
